# Supplementary material for: Chromothripsis during telomere crisis is independent of NHEJ, and consistent with a replicative origin
Source: Genome Res. 2019 May;29(5):737–49. doi: 10.1101/gr.240705.118 (PMC6499312; doi:10.1101/gr.240705.118)
Supplement: Supplemental Material [file supp_gr.240705.118_Supplemental_file_1.zip › contigs/annotated_contigs/DB55/contig.4.DB55_length_852_mean_cov_7.00821596244.docx]

**DB55_length_852_mean_cov_7.00821596244**

```
AATGGAATTGAATGGAGACTAATGGGATAGAATCAAATGGAATGGCATCGAATGGAATGGA|CTCGAATGCAGTGTG|CTCGAATGGAA
 >chrY:13801064-13801125 + E=8e-16 p=0e+00                                   >chr1:2242
TGGAATCGAATGGATTGACA|T|TCAGGTTTGGGCAGAATCAGGCGCATGACCCTTAAGATATCAAACCAAGTTCGCCTGAAGCCCAGA
03930-224203962 + E=2e-04 >chr7:154585155-154585482 + E=8e-185
GCCATGAACCAATTTCCCTAATTAGCTAGAGAGTGTCCCTGGGTATTTTATCTTTAAGGAGGAGGAATTAGAGAATGGAGCCTTCAACA

GCCCAGGCTGGGATGCTTCTCACCCTGTGTCGCTCTCTGGGGCCTCCCGCCAGCCCTTTTCCGGTTCTTTCTCTGTCCCGCCTCCCCGG

ACCCCTCGCTGCCCGGTCAGGTGGTTAGTTGTGCGTTTGCCTGGTACTGCGGTGTGAAAGTCAAGGCATCAGCTGGAGAGAC|AGGGGA
                                                                                    >chr
GACGGGTGAAGAACCGATGGTCAGACGCGCCCGAGGTGGCGCCAGAGTCGTCTCAGCCCTCGGGCCTGGGACTTGGGGACCGGCCGCCC
7:154585934-154586352 + E=1e-239
AGCTGCTGCGGGGAGCCCGCGGCGCAGAGTCCCGGCTCCGGCGTGGCTGCCACCTCGTGGCTGGTGCAGGCGCTGCCGCAGACCCGACC

GAACCCATCCGTGAATTGCCAGAGGCGCCCCGTGCCTGGCACCTGGACGCCGTCCGCAGTTCACTGAGTGTTGCTTCCCTTTATCCGCG

CTCACCGCTGTGACCTAAGTGAGCGTCCTCCTCGTTGCAGCTGATGCTCAGAGAGGAGGGGCCCTGCCTTTCCCCAGACCCCGCCGACA

GCCTCCCCCAGCGGGCTCCAGGCGTGCGTCCCTCCCCACGTCCTGGTGAGGCCAGC
```
